# Editorial: Frailty- and age-associated diseases: possibilities for intervention (Volume 2)

**DOI:** 10.3389/fragi.2025.1759325

**Published:** 2026-01-07

**Authors:** Consuelo Borrás, José Viña, Cristina Mas-Bargues

**Affiliations:** Freshage Research Group, Department of Physiology, Faculty of Medicine, University of Valencia, Centro de Investigación Biomédica en Red Fragilidad y Envejecimiento Saludable-Instituto de Salud Carlos III (CIBERFES-ISCIII), INCLIVA, Valencia, Spain

**Keywords:** age-associated diseases, aging, biomarker, frailty, intervention

## Introduction

Frailty and age-associated diseases represent a growing challenge for healthcare systems worldwide as populations age at unprecedented rates. Sarcopenia, cognitive impairment, osteoporosis, stroke-related complications, and chronic metabolic diseases reduce the physiological reserve, increase the vulnerability to stressors, and accelerate the functional decline. This second volume of the *Frailty- and Age-Associated Diseases* Research Topic brings together twelve complementary contributions that collectively highlight emerging opportunities for risk stratification, early identification, and intervention in older adults. From functional and biological biomarkers to multicomponent interventions and methodological roadmaps, this Research Topic provides a multidimensional overview of the current and future directions in frailty research.

## Main contributions to the Research Topic

A first group of articles focuses on assessment and biomarker screening, emphasizing the need for reliable, simple, and scalable tools and biomarkers to support clinical decision-making. Sun et al. demonstrate that handgrip strength, an accessible, low-cost, and functional measure, is strongly associated with all-cause mortality in individuals with low bone mass, underscoring its utility as a routine screening tool (Sun et al). Complementing this, a narrative synthesis of frailty scales provides clinicians with a practical overview of existing instruments and their applicability across settings. Additional contributions explore nutritional and metabolic biomarkers, such as folate-related indicators, and their associations with cognitive impairment (Lv et al), as well as a meta-analysis linking cognitive frailty to increased fall risk (Liu et al). Together, these studies converge on a key message: early identification of frailty requires both functional metrics and biologically informed markers (Wang et al).

A second set of studies examines interventions aimed at mitigating frailty progression or improving outcomes. A *post hoc* analysis of a large cohort of older adults with sarcopenia shows that multicomponent interventions (combining exercise, nutrition, and behavioral support) can extend institutionalization-free survival, demonstrating the value of integrated approaches (Ji et al). Preclinical work evaluating high-intensity interval training (HIIT) in middle-aged mice further highlights the potential for exercise to improve both physical and cognitive parameters (Stephenson et al). Deng et al. present a network meta-analysis investigating acupuncture-based therapies for postmenopausal osteoporosis, where several modalities appear to enhance bone health when combined with standard treatment (Deng et al). Despite methodological heterogeneity, these findings encourage continued exploration of combined lifestyle, rehabilitative, and complementary interventions.

Other contributions explore the underlying mechanisms and contextual factors, offering a bridge between biology and clinical practice. A review of the microbiota–aging axis in sarcopenia suggests that gut dysbiosis contributes to muscle decline and may become a therapeutic target in the future (Cheng et al). Moreover, a comprehensive epidemiological analysis of dementia and cognitive impairment emphasizes the intertwined roles of frailty, malnutrition, and healthcare utilization, illustrating the complexity of managing multimorbidity in older adults (Merchant et al). Indeed, two studies on sarcopenia in type 2 diabetes mellitus highlight the importance of rigorous methodology when studying high-risk subgroups (Whaikid et al; Xu et al). Finally, Wang et al. provide an in-depth review of post-stroke dysphagia, identifying substantial gaps in diagnostic consistency, treatment protocols, and inclusion of patients with cognitive impairment; thereby mapping a clear agenda for future rehabilitation research (Wang et al).

## Convergencies and discrepancies

Across these diverse studies, several common themes emerge. First, frailty is reaffirmed as a multidimensional syndrome that requires integrated approaches. Second, there are simple screening tools with high clinical value (e.g., handgrip strength, standardized scales). Third, multicomponent interventions (exercise + nutrition + rehabilitation) are the most promising strategies for preserving independence and avoiding institutionalization. Fourth, biological markers involving nutrition, metabolism, and the gut microbiota offer promising translational opportunities and could help refine risk stratification and personalized intervention.

At the same time, the Research Topic highlights persistent methodological challenges. Several studies emphasize variability in definitions and endpoints, study designs with a risk of bias (e.g., lack of blinding or insufficient sample size), exclusion of relevant subgroups (e.g., individuals with cognitive impairment), and the scarcity of longitudinal studies that include hard outcomes (mortality, institutionalization). Reproducibility is particularly limited in areas such as dysphagia research, complementary medicine trials, and microbiota-related interventions. Moreover, translating preclinical results (e.g., HIIT in animal models) warrants caution before producing clinical recommendations.

## Conclusion

In conclusion, this Research Topic provides a rich and multidimensional view of frailty and age-associated diseases, integrating clinical, biological, and rehabilitative perspectives. The collection highlights both the progress made and the substantial opportunities that remain. Advancing the field will require harmonized methodologies, translational studies linking mechanisms to interventions, and a continued commitment to preserving independence and quality of life in the aging population.

